# Supplementary material for: Prediction of on-field rugby scrummaging contact forces from videos using artificial neural networks
Source: PLoS One. 2026 Apr 8;21(4):e0330097. doi: 10.1371/journal.pone.0330097 (PMC13061245; doi:10.1371/journal.pone.0330097)
Supplement: Attachment — Submitted filename: Letter.pdf [file pone.0330097.s002.pdf]

Critical Review Letter – Manuscript PONE-D-25-40825

Title: "Prediction of on-field rugby scrummaging contact forces from videos using Artificial Neural Networks".

Dear Researchers,

Thank you for the invitation to review this manuscript. The study presents an innovative proposal of high clinical and sporting relevance: predicting contact forces in the rugby scrum using only video kinematics and Recurrent Neural Networks (RNNs). The potential for cervical injury prevention is promising. However, after a detailed analysis of the submission, I have identified critical issues and <u>severe ethical and editorial failures</u> that fundamentally compromise the validity, reproducibility, and compliance of the work.

Final Recommendation: Rejection with the possibility of resubmission only after substantial revision and proof of compliance with editorial policies.

The severity of the flaws is categorized below:

A. Ethical and Editorial Non-Compliance (Fatal Flaws)

1. **Severe Violation of the Open Data Policy (PLOS ONE):** The authors explicitly state: "The data underlying the results presented in the study are not available. Data are partially owned by a third party and authors do not have permission to share the data." This violates the fundamental requirement of PLOS ONE that all underlying data be made available without restriction upon publication. The justification of "third-party ownership" is unacceptable for a study seeking to contribute to open science. The reproducibility of the work is, therefore, impossible.
2. **Contradiction and Failure in the Ethics Statement:** The manuscript marks **"N/A"** in the Ethics Statement section, implicitly claiming not to involve human participants. However, the Methods section confirms the use of data from **176 elite players** with **written informed consent** and approval from the University

of Bath Ethics Committee. The omission or deliberately incorrect statement of ethical approval is a serious breach of editorial integrity.

B. Methodological and Technical Flaws (ML and Biomechanics)

3. **Questionable Data Augmentation Methodology (GAN/Mixup):** The use of a Generative Adversarial Network (GAN) to create 1,000 synthetic force signals in a safety context has not been adequately justified in terms of **physical validity**. The high statistical correlation between generated and measured data (R=0.99) does not guarantee that the synthetic forces respect the biomechanical laws of a human collision. The risk of the trained model learning **synthetic artifacts** from the GAN, instead of real patterns, is high and dangerous for an application aimed at preventing injuries.
4. **Non-Scalable Methodology:** The model relies on **manual 2D labeling** of the C7 and Lumbar landmarks. This makes real-time, on-field (*in-game*) application unfeasible and burdensome. The authors do not provide: a) A labeling protocol, b) Inter-rater reliability metrics (essential for validating the kinematic input), or c) A validated automated *pose estimation* model to replace manual labeling.
5. **Clinically Unacceptable Variability in Results:** Despite the low normalized RMSE (9.3%), the Bland-Altman analysis reveals **extreme Limits of Agreement** (–530 N to +640 N). In a high-energy collision scenario, this margin of error in peak force prediction is **clinically unacceptable** and can mask the difference between a safe scrum and one with a high risk of cervical injury.
6. **Omission of Critical Biomechanical Covariates:** The model ignores the **individual body mass** of the players as an input variable. Contact forces in a collision are a direct function of mass and acceleration (F=ma). The exclusion of this fundamental biomechanical variable compromises the model's robustness and is a likely cause of the high variability observed in the Bland-Altman analysis (Point 5).

C. Presentation and References

7. **Exaggerated Conclusion:** The claim that the method "could revolutionize player monitoring" is highly speculative. The unacceptable clinical variability (± 640 N), the failure of scalability (manual labeling), and the dependence on non-sharable

data prevent the work from being considered a transformative tool in its current state.

8. **References (Requires Review):** Excessive self-citation (20% of the total) and the use of conference proceedings (e.g., Pomarat et al., [39]) to substantiate methodological arguments were identified, which weakens the theoretical basis of the submission.

Summary: The study has significant intellectual potential but is irremediably compromised by ethical failures (Data and Consent) and methodological flaws (Scalability and Clinical Validity of Error). **Rejection is the only possible recommendation.**
