# Supplementary material for: Prediction of on-field rugby scrummaging contact forces from videos using artificial neural networks
Source: PLoS One. 2026 Apr 8;21(4):e0330097. doi: 10.1371/journal.pone.0330097 (PMC13061245; doi:10.1371/journal.pone.0330097)
Supplement: Attachment — Submitted filename: Reviewer_comments.docx [file pone.0330097.s004.docx]

**Revision required [PONE-D-25-40825] - [EMID:18fa6022957bc0ab]**

**Prediction of on-field rugby scrummaging contact forces from videos using Artificial Neural Networks**

**Response to Editors and Reviewers**

We thank the editors and reviewers for providing feedback on our paper. We trust that changes resulting from your comments have improved the quality of this manuscript. Changes to the manuscript have been highlighted in red.

**Reviewer Comments:**

**Reviewer #1**

*This research implementing machine learning technique to predict the injury risk in the high-risk sports, rugby, to provide further information for team staff to avoid injury risk. I have several comments as below:*

*1. From line 55-71, authors describe the traditional data collection shall be improve by implementing wearable technology (WT) include GPS and IMU. However, it seems that this article tried to improve the prediction performance by integrating video-analysis techniques. I suggest to revise this paragraph to enhance the importance of implementing video-based analysis instead of highlights the WT importance.*

***Author response:*** In this paragraph, we described the current use of traditional sensors and wearables, highlighting their drawbacks (including comfort issues and measurement inaccuracies). However, we agree that a deeper explanation of the importance of the video-based analysis is needed. Therefore, to emphasize the advantages of video-based analysis as the main focus of the study, the final part of this paragraph has been revised to read as follows:

“This method could involve the use of video-based motion analysis to allow the remote quantification of player kinematics without physical sensors. This enables a fully non-invasive estimation of body positions, velocities during real-game situations. Therefore, implementing video-derived kinematic features as input to artificial neural networks (ANNs) represents a powerful alternative to wearable systems for estimating contact forces in rugby scrummaging.”

*2. From line 80, authors raise the predict target GRF. However, it shall be clarified that GRF is stand for “Ground Contact Forces” or “Ground Reaction Forces.”*

***Author response:*** Thank you for this suggestion. We have clarified the term at its first appearance as follows:

“Furthermore, ANNs have been used to predict ground reaction forces (GRF) based on kinematics…”

*3. On line 56-59, authors illustrate that the traditional methods “miss forces transferred through neck and head…..” However, from my point of view, this research also used the sensor installed on player’s shoulder. How does this work is different from other research?*

***Author response:*** We appreciate this opportunity to clarify. The previous study by Cazzola et al. [13] indeed used shoulder-mounted pressure sensors to measure the normal contact force. Our model estimates the same shoulder contact forces without using any physical sensors, relying solely on video-derived kinematic data. However, it is true that we do not measure neither estimate forces transferred through neck and head. Therefore, the sentence “these methods rely on limited sensor resolution, and they also may miss forces transferred through neck and head” was deleted.

*4. On line 97-111, dataset shall be describe as “how many total dataset had been detected.” That is, how many scrummaging had been performance by the 176 elite rugby players to produce the numbers of data collected.*

***Author response:*** Thank you for pointing this out. We have clarified the dataset size in the Dataset section to specify the total number of scrummaging trials:

“Each team (two forward packs, referred to as ‘Team A’ and ‘Team B’) performed between 12 and 16 scrums (resulting in a total of 330 scrummaging trials) using…”

*5. Continued with No.4, if the dataset had been modified, what is the reason for implementing GAN to increase the data size?*

***Author response:*** We thank the reviewer for this question so we can clearify it. No modification is mentioned in the paper. Data were only processed (filtered, derived, normalized...) and the only “modification” was the removing of “outliers”. The implementation of a GAN was to augment the original dataset in order to improve the ANN´s generalization and robustness. This is because the original dataset had limited number of trials to train an ANN with this type of highly nonlinear dynamic data resulting from perturbations (impact forces during rugby scrummaging). We have revised the Data augmentation section to clarify this as follows:

“….the original dataset. Therefore, the GAN was trained to synthesize additional realistic velocity–force pairs following the same statistical distribution as the measured data. This data augmentation was implemented to balance the dataset and try to reduce overfitting due to the limited number of real scrum trials, not to modify or substitute the original dataset. The generator was….”

*6. The variables used in this research shall be identified. This research aims to predict the injury risk. However, using of variables represents “injury risk” is not clear enoug.*

***Author response:*** We appreciate the reviewer’s comment about the variables that determine the injury risk. However, this research does not aim to predict the injury risk. In the Data Analysis section the variables used (linear velocity derived from the position coordinates of the midpoint between the C7 and lumbar locations and the normal contact forces) were described. Therefore, the model does not directly predict “injury risk,” it predicts scrummaging shoulder contact forces, which could be used as overloading biomechanical indicators associated with potential injury risk. This is the message stated through the whole paper: the predicted contact forces can be used as indicators of injury risk based on their magnitude.

**Reviewer #2**

*1.* Methods detail and replicability.

The description of manual labeling of C7 and lumbar landmarks is insufficient. It is essential to specify the number of raters involved, whether inter- or intra-rater reliability was assessed, and the software utilized, as manual labeling is susceptible to error. Data cleaning is mentioned, but the number of trials excluded and the justification for the chosen thresholds are not reported. Without this information, it is challenging to assess whether exclusions biased the dataset. The GAN and Mixup approaches are introduced but not described in sufficient detail for replication. A clearer specification of the GAN architecture and training procedure, as well as the parameters used in Mixup, is necessary.

***Author response:*** We thank the reviewer for highlighting the need for more detail. Here the response.

Reliability of the labelling data was assessed and was high, as reported in Preatoni et al. (2012). This information has been added to the manuscript as follows: “The two-dimension (2D) coordinates of the C7 vertebra and lumbar locations of the front row players were manually labelled from the provided top-view videos. Reliability of these labelled data was assessed and was high, as reported by Preatoni et al. [41]. In addition….”

Regarding data cleaning and trial exclusions, 42 trials were finally used as the rest of trial were excluded because the velocity and contact force peak values were outside the ranges of 2.5–4.5 m·s⁻¹ and 1–4 kN, respectively. These thresholds were chosen based on both physiological plausibility and prior literature in rugby scrums (Cazzola et al., 2015). Trials beyond these ranges were also inspected and found to result from technical execution errors during data collection, such as sensor misalignment. This explanation has been added to the paper as follows: “A total of 42 trials were finally selected from the original dataset after data cleaning, which involved removing empty files and scrum trials in which velocity and contact force peak values were outside the range of 2.5-4.5 m·s^-1^ and 1-4 kN, respectively. Based on the literature [13], values beyond these thresholds were considered physiologically unrealistic or potentially indicative of technical execution errors during the trials, such as sensor misalignment.”.

In the “Data augmentation” subsection, the GAN architecture and hyperparameters are explained in detail, as follows: “The generator was a sequential model with a latent dimension of 50 and composed of one dense layer (26x128 neurons) followed by two convolutional layers (64 and 2 neurons, SELU and tangential activations) ending with one dense layer (202 neurons, tangential activation). Batch normalization layers were implemented in the generator model. The discriminator was a sequential model composed of two convolutional networks (64 and 128 neurons, LeakyReLU activation) and one dense layer (one neuron, sigmoid activation). In addition, layers of dropout were added to regularize the discriminator with a dropout rate of 40%. The convolutional layers had a kernel size of 5 and a stride of 2 with zero padding. The selection of the GAN´s architecture and its hyperparameters relied on manual tuning, according to the correlation and root mean squared error (RMSE) between its generated curves and the measured ones.”

Regarding Mixup, the specification of the Mixup coefficient was added as follows: “In addition, using the Mixup technique [42], data were augmented to enlarge the input dataset of the recurrent neural network (RNN). Mixup is a non-generative data augmentation approach that was used to create new velocity–force signal pairs by linearly combining pairs of temporally normalized velocity and force time-series. To construct physically meaningful intermediate time-series while preserving temporal structure, a constant mixup coefficient of 0.5 was used. This improves the generalization of the RNN as it provides synthetic data that decreases overfitting.”

*2. Limitations of 2D data.*

*Although the limitation of relying on a single top-view camera is acknowledged, this point warrants more explicit discussion. Important postural information is inevitably lost in 2D, which could directly account for some of the observed prediction variability.*

***Author response:*** We agree with this point regarding the use of 2D data. However, although this implies a limitation in terms of obtaining movement information, such information is less relevant to the model because, in rugby scrummaging, the depth component of movement is notably smaller in magnitude than the components within the plane of movement. Therefore, the explanation about this point has been added to the manuscript as follows:

“This approach resulted in a loss of information regarding the player’s poses during the scrum, which could be highly relevant to evaluate the correlation between player kinematics and scrum contact force generation. However, in rugby scrummaging, players are trained and conditioned to maximize forward (horizontal) force production while maintaining stable body positions, with vertical force and movement components playing a secondary role in the overall movement strategy [62]. Nevertheless, there is considerable room for improvement….”

*3. Prediction variability.*

*The Bland–Altman plots demonstrate wide limits of agreement (approximately ±600 N). This is not trivial, particularly when considering injury risk thresholds. While the average error is low, a clearer discussion of the practical implications of such variability would benefit readers—for instance, whether the tool is more suited for monitoring trends across groups rather than making judgments on individual events.*

***Author response:*** Thank you for this valuable suggestion. We have extended the discussion about the implications of the observed variability:

“Shoulder contact forces come from the collision between two soft tissues in movement, which could add more variability into the force signal. This could be a reason for the results observed in the Bland–Altman plot, whose limits of agreement (±600 N) indicate a notable variability. However, this represents approximately 15% of the maximum measured peak force. Therefore, the model could be better suited for group-level monitoring and trend analysis (e.g., detecting systematic increases in impact load or comparing engagement techniques) rather than for making clinical or safety judgments on single events and individual predictions. Thus, despite scrummaging being a challenging task….”

*4. Automated pose estimation.*

*The manuscript suggests eventual automation via pose estimation, yet the challenges of this transition are not discussed. It is important to comment on the likely decrease in accuracy when transitioning from carefully labeled manual landmarks to automatically detected ones, and how this issue might be addressed.*

***Author response:*** We appreciate this insightful comment and have expanded the Discussion to explicitly address the challenges and implications of using manual landmark labeling or automated pose estimation.

“However, it must be considered that the transitioning from manually labelled landmarks to automated pose estimation introduces additional sources of error related to landmark misidentification, depth ambiguity, and occlusion. Menychtas et al. [63] have shown that 2D pose estimation algorithms yield accurate estimates for large joint movements but struggle to capture small, high-frequency motions or those occurring out of plane. Similarly, automated vision-based tracking systems may exhibit greater variability when the visibility of landmarks is compromised [64]. On the other hand, manual labelling is more consistent, but it could introduce a static offset due to a user´s bias [63]. To mitigate these limitations, future work should employ confidence-weighted filtering to disregard low-certainty keypoints, use temporal smoothing to correct transient errors, and train the pose estimation models using hybrid datasets combining automatically estimated landmarks and manual corrections. These strategies can improve robustness and help bridge the accuracy gap between manual and automated tracking systems. Nevertheless, there is still a significant time-saving benefit of the automated algorithms over manual annotation [63]. This efficiency facilitates the annotation of a larger number of body landmarks and, consequently, incorporating this richer information as input to the neural network…”

*5. The Generalizability.*

*The dataset utilized in this study is derived from elite male rugby teams. It is pertinent to briefly consider the extent to which the model may be applicable to other cohorts, such as youth, amateur, or female players, where variations in anthropometry and technique are evident.*

***Author response:*** We acknowledge this point and have added the following clarification to the Discussion:

“These machine learning models employed in prior research can provide the time of impact and the player’s posture, which are the inputs required for applying our scrum engagement force prediction model. However, as the used dataset to train this model consisted exclusively of elite male rugby players, its generalizability to other populations (e.g., female, youth, or amateur players) remains untested. These groups differ in anthropometry, strength, and scrummaging technique. Nevertheless, as the inputs of our model are solely linear velocities, its predictions should not be strongly affected by players' physical differences. Even so, future work should include additional data from diverse cohorts to enable transfer learning and ensure model applicability across playing levels.”

*6. Presentation Issues.*

*The tables and figures are generally well-presented; however, it is advisable to include units and sample sizes in the captions where relevant. Additionally, Table 2 appears to have a formatting issue in the A3-R row, which requires correction. It may also be beneficial to incorporate a small set of example traces (best, average, worst prediction) to demonstrate the model's performance under varying conditions.*

***Author response:*** We appreciate this feedback and we have implemented the corrections.

**Reviewer #3**

*1. Severe Violation of the Open Data Policy (PLOS ONE): The authors explicitly state: “The data underlying the results presented in the study are not available. Data are partially owned by a third party and authors do not have permission to share the data.” This violates the fundamental requirement of PLOS ONE that all underlying data be made available without restriction upon publication. The justification of "third-party ownership" is unacceptable for a study seeking to contribute to open science. The reproducibility of the work is, therefore, impossible.*

***Author response:*** Data will be available in <https://doi.org/10.5281/zenodo.18048888>. It will contain the data that were used to train the RNN (those coming from the GAN and the Mixup) and the 42 trials from the original dataset that were used to evaluate the predictions. Moreover, the GAN and the RNN models along a code to use them will be available as well.

*2. Contradiction and Failure in the Ethics Statement: The manuscript marks "N/A" in the Ethics Statement section, implicitly claiming not to involve human participants. However, the Methods section confirms the use of data from 176 elite players with written informed consent and approval from the University of Bath Ethics Committee. The omission or deliberately incorrect statement of ethical approval is a serious breach of editorial integrity.*

***Author response:*** This was a misunderstanding as the data used in the present study were collected for other previous studies so we did not need any specific ethic approval for our study. However, in the “Dataset” subsection of our study we stated that: “Data collection from these participants was carried out following the institutional ethics committee at the University of Bath, School for Health Research Ethics Approval Panel, and after individual written informed consent was provided by each participant.” Therefore, this issue will be amended in the PLOS ONE submission platform.

The study was granted by an institutional ethics committee at the

University of Bath, School for Health Research Ethics Approval Panel.

*3. Questionable Data Augmentation Methodology (GAN/Mixup): The use of a Generative Adversarial Network (GAN) to create 1,000 synthetic force signals in a safety context has not been adequately justified in terms of physical validity. The high statistical correlation between generated and measured data (R=0.99) does not guarantee that the synthetic forces respect the biomechanical laws of a human collision. The risk of the trained model learning synthetic artifacts from the GAN, instead of real patterns, is high and dangerous for an application aimed at preventing injuries.*

***Author response:*** Thanks for highlighting this point, which allowed us to clarify it. The main reason for performing data augmentation was to increase the size of the training dataset and reduce the risk of model overfitting. We agree that correlation alone does not guarantee the physical validity of the synthetic data. Therefore, apart from correlation values, we presented the RMSE values as well (Data Augmentation subsection). In this way, we ensured that the patterns of the synthetic data (linear velocities and contact forces) were similar to those of the real measured data, both in shape and magnitude. In addition, to avoid or minimize the learning of artifacts and biases from GAN-generated data by the model, two techniques were applied: 1) a deep convolutional GAN architecture was used and 2) mini-batch discrimination was used during training. In addition, another technique (Mixup technique) different from the GAN was used to create physically meaningful intermediate time-series while preserving temporal structure. In this way, both strategies contribute to reduce overfitting. The GAN-related techniques aim to prevent artifact learning by improving the quality and diversity of synthetic data, whereas Mixup directly regularizes the RNN by forcing it to learn smooth temporal relationships, preventing abrupt or trial-specific variations in the predicted signals.

*4. Non-Scalable Methodology: The model relies on manual 2D labeling of the C7 and Lumbar landmarks. This makes real-time, on-field (in-game) application unfeasible and burdensome. The authors do not provide: a) A labeling protocol, b) Inter-rater reliability metrics (essential for validating the kinematic input), or c) A validated automated pose estimation model to replace manual labeling.*

***Author response:*** We appreciate this feedback regarding the use of the model in real-world applications. As the reviewer noted, the model was trained using velocity data derived from the 2D labelled coordinates of C7 and lumbar landmarks. However, it is not mandatory to do manual labelling of the landmarks to use the model as its input is the linear velocity. Therefore, one can calculate the velocity using a less time-consuming technique such as automatic pose estimation models. However, there is no specific pose estimation model designed to estimate the same 2D point coordinates as those used to calculate the linear velocities that our force estimation model uses as input. Therefore, users are encouraged to experiment with different pose estimation models when our model becomes freely available.

Regarding the labeling protocol and inter-rater reliability metrics, as it was answered to Reviewer 2 Comment #1, reliability of the labelling data was assessed and was high, as reported by Preatoni et al. (2012). This information has been added to the manuscript as follows: “The two-dimensional (2D) coordinates of the C7 vertebra and lumbar locations of the front row players were manually labelled from the provided top-view videos. Reliability of these labelled data was assessed and was high, as reported by Preatoni et al. [41]. In addition….”

*5. Clinically Unacceptable Variability in Results: Despite the low normalized RMSE (9.3%), the Bland-Altman analysis reveals extreme Limits of Agreement (–530 N to +640 N). In a high-energy collision scenario, this margin of error in peak force prediction is clinically unacceptable and can mask the difference between a safe scrum and one with a high risk of cervical injury.*

***Author response:*** Yes, it is true that the prediction variability would be too high for a clinical application. However, the forces predicted by our model can still be useful for identifying general trends in group-level monitoring and for informing safety-related decisions in rugby scrummaging. Accordingly, considering this comment and a similar suggestion from Reviewer 2, we have extended the discussion as follows:

“Shoulder contact forces come from the collision between two soft tissues in movement, which could add more variability into the force signal. This could be a reason for the results observed in the Bland–Altman plot, whose limits of agreement (±600 N) indicate a notable variability. However, this represents approximately 15% of the maximum measured peak force. Therefore, the model could be better suited for group-level monitoring and trend analysis (e.g., detecting systematic increases in impact load or comparing engagement techniques) rather than for making clinical or safety judgments on single events and individual predictions. Thus, despite scrummaging being a challenging task….”

*6. Omission of Critical Biomechanical Covariates: The model ignores the individual body mass of the players as an input variable. Contact forces in a collision are a direct function of mass and acceleration (F=ma). The exclusion of this fundamental biomechanical variable compromises the model's robustness and is a likely cause of the high variability observed in the Bland-Altman analysis (Point 5).*

***Author response:*** We did not include players’ masses as inputs to the model. The reason is that, in rugby scrummaging, players within each team push forward simultaneously, and our data do not allow us to determine what proportion of the total measured force is generated by each individual player. Moreover, players do not contribute equally in every scrum collision. Consequently, including individual masses will likely introduce noise into the predictive model rather than provide information. In addition, as mentioned in the paper: “Although our RNN did not consider the mass of the players, this would not be a weakness of the RNN since Quarrie and Wilson [56] found that, even though the mean correlation between players pack mass and the scrum force was higher than 0.8, the 95% confidence interval of this correlation included zero making this relationship variable. In addition, it should be noted that heavier forward packs generate greater impact force only in the case of the full scrum technique [57].”

*7. Exaggerated Conclusion: The claim that the method "could revolutionize player monitoring" is highly speculative. The unacceptable clinical variability (± 640 N), the failure of scalability (manual labeling), and the dependence on non-sharable data prevent the work from being considered a transformative tool in its current state.*

***Author response:*** All the points raised in this comment have been addressed in the previous responses. We therefore hope that these revisions have improved the quality of the paper and that the model developed in this study may serve as a useful first step toward revolutionizing rugby player monitoring.

*8. References (Requires Review): Excessive self-citation (20% of the total) and the use of conference proceedings (e.g., Pomarat et al., [39]) to substantiate methodological arguments were identified, which weakens the theoretical basis of the submission.*

***Author response:*** We thank the reviewer for this comment. It is true that we citated some papers (now < 16%) whose authors are co-authors of this study. However, there are few studies related to rugby biomechanics in which contact forces are measured. In addition, the data used in our study originate from previous work conducted by the same research group. We would welcome the opportunity to cite more studies on rugby biomechanics conducted worldwide; however, to the best of our knowledge, we have cited all relevant and suitable studies available for the purposes of our work.

On the other hand, we would like to clarify that the number of conference proceedings used in the Methods section is limited and carefully justified. These references correspond to the Adam optimizer (Kingma and Ba, 2015) and the mixup method (Zhang et al., 2017). They are both cited using their original and canonical references, which are conference/arXiv publications that remain the standard citations for these widely adopted methods. In addition, the study of Pomarat et al., apart from the Introduction, was cited in the Discussion section to contextualize the results rather than to substantiate methodological arguments. Nevertheless, the citation of Pomarat has been replaced by its journal version. We have reviewed the manuscript to ensure that methodological and theoretical claims are supported by appropriate peer-reviewed journal references wherever possible.
